# Assessment of Reticulocyte Hemoglobin Content in Patients Undergoing Chronic Hemodialysis for Optimized Anemia Management

**DOI:** 10.7759/cureus.83867

**Published:** 2025-05-10

**Authors:** Salma Daali, Kenza El Atifi, Nabil Hamouche, Zineb Aboudar, Mona Jabrane, Amina Bendriouich, Ayoub Rafei, Sara Mazighi, Samia Elkarci, Siham Aboulmakarim, Wafaa Fadili, Sanae Sayagh, Mariam Chettati, Inass Laouad

**Affiliations:** 1 Nephrology, Centre Hospitalier Universitaire Mohammed VI, Marrakesh, MAR; 2 Nephrology, Faculty of Medicine and Pharmacy in Marrakech, Cadi Ayyad University, Marrakesh, MAR; 3 Hematology, Centre Hospitalier Universitaire Mohammed VI, Marrakesh, MAR; 4 Hematology, Faculty of Medicine and Pharmacy in Marrakech, Cadi Ayyad University, Marrakesh, MAR; 5 Biochemistry, Centre Hospitalier Universitaire Mohammed VI, Marrakesh, MAR; 6 Biochemistry, Faculty of Medicine and Pharmacy in Marrakech, Cadi Ayyad University, Marrakesh, MAR

**Keywords:** anemia management, chronic hemodialysis, diagnostic biomarkers, iron deficiency, reticulocyte hemoglobin content

## Abstract

Introduction

Anemia is a common complication in patients undergoing hemodialysis, requiring accurate assessment for effective management, particularly due to its often multifactorial origin. Among the available biomarkers, reticulocyte hemoglobin content (Ret-Hb) has emerged as a promising tool for evaluating iron availability and the efficacy of erythropoietin (EPO)- and iron-based therapies.

Objectives

This study aims to assess the clinical utility of Ret-Hb measurement in patients undergoing chronic hemodialysis and to compare its performance with conventional biochemical markers of iron metabolism (serum ferritin, serum iron, transferrin) in the diagnosis of iron deficiency.

Materials and methods

A prospective observational study was conducted on hemodialysis patients from the region over a six-month period, from March to August, 2024. The study was carried out within the Department of Nephrology, Hemodialysis, and Renal Transplantation of Centre Hospitalier Universitaire Mohammed VI, Marrakesh, Morocco, in collaboration with the Biology Laboratory. The parameters analyzed included demographic, clinical, biological, and therapeutic data. Data analysis involved the use of Spearman correlation coefficients with a significance threshold set at 0.05, and receiver operating characteristic (ROC) curve analysis to evaluate the diagnostic performance of different parameters. Data entry was performed using Excel, and statistical analyses were conducted using MiniTab (Minitab, LLC, State College, Pennsylvania, United States) and MATLAB (The MathWorks, Inc., Natick, Massachusetts, United States) software.

Results

A total of 105 patients were enrolled, with a mean age of 50.4 ± 16.3 years and a male-to-female sex ratio of 0.3. According to the complete blood count, anemia was identified in 87 (82.8%) patients, predominantly of the normochromic normocytic type. The mean Ret-Hb level was 30.03 ± 8.1 pg. A Ret-Hb value below 28 pg was observed in 32 (30.4%) patients. Additionally, 75 (71.4%) patients exhibited a transferrin saturation coefficient below 20%, and 55 (52.3%) presented with a serum ferritin level below 200 ng/mL. Analysis revealed that Ret-Hb had a sensitivity of 90% and a specificity of 87.5% for diagnosing iron deficiency, confirmed by a significant ROC curve (area under the curve (AUC) = 0.81). Moderate correlations were observed between Ret-Hb and ferritin levels (r = 0.59), as well as between Ret-Hb and transferrin saturation (r = 0.52).

Conclusion

Ret-Hb proved to be a sensitive and specific marker for the diagnosis of iron deficiency in hemodialysis patients. This study highlights the potential role of Ret-Hb in monitoring anemic patients undergoing hemodialysis and assessing their response to iron supplementation.

## Introduction

Chronic kidney disease (CKD), due to its multiple complications, constitutes a major public health concern [1]. At stage 5 of CKD, the initiation of renal replacement therapy becomes necessary [2]. In Morocco, the number of patients undergoing chronic hemodialysis currently exceeds 35,000, compared to an estimated 32,000 in 2020 [3].

Anemia constitutes a severe and common complication in CKD, particularly evident in end-stage renal disease (ESRD). It is a major contributor to morbidity, mortality, and diminished quality of life in this patient population, affecting 60-80% of individuals [4]. Anemia in CKD primarily results from inadequate erythropoietin production, reduced red blood cell lifespan, frequent blood losses, and the presence of a chronic inflammatory state [4].

The management of anemia in CKD requires rigorous monitoring using specific biological parameters to ensure appropriate therapeutic interventions tailored to each patient. In patients with ESRD, the frequent coexistence of inflammation and iron deficiency complicates the etiological diagnosis of anemia. Traditional biomarkers of iron status are often altered in the setting of inflammation, limiting their ability to accurately reflect the body's iron stores [5]. In chronic hemodialysis patients, distinguishing between inflammatory anemia and mixed anemia (inflammatory and iron-deficiency) is particularly challenging. The conventional iron profile is disrupted in the context of inflammation and fails to reliably assess iron status [5].

Currently, reticulocyte hemoglobin content (Ret-Hb) is emerging as a promising indicator. Ret-Hb measures the hemoglobin content of reticulocytes, providing a reasonably accurate reflection of iron bioavailability in the bone marrow [6]. This allows for a better evaluation of the response to iron supplementation and the administration of erythropoiesis-stimulating agents. Thus, the aim of this study is to evaluate the diagnostic value of measuring the automated hematological parameter, Ret-Hb, in chronic hemodialysis patients, compare it with conventional biochemical markers of iron metabolism (e.g., serum ferritin and transferrin saturation), and assess its performance in diagnosing iron deficiency

## Materials and methods

Study design

This was a prospective observational study conducted over six months, from March 2024 to August 2024. It focused on evaluating the utility of the automated hematological parameter, Ret-Hb, for diagnosing iron deficiency in chronic hemodialysis patients.

Study setting 

The study was carried out within the Department of Nephrology, Hemodialysis, and Renal Transplantation at Centre Hospitalier Universitaire (CHU) Mohammed VI, Marrakesh, Morocco, in collaboration with the institution's Hematology and Biochemistry Laboratories. Patients were recruited from various hemodialysis centers in the Marrakesh region.

Exclusion and inclusion criteria

The study sample was randomly recruited from a population of individuals undergoing chronic hemodialysis. Eligible participants included adult patients aged 18 years or older who had been receiving hemodialysis treatment for a minimum duration of three months and provided written informed consent. Patients were excluded if they had a recent history of clinically significant bleeding or had received blood transfusions, due to the potential interference with hematological assessments. Individuals with obvious signs of inflammation, active infectious diseases, or malignancies were also excluded because these conditions can influence iron metabolism and related biomarkers.

Laboratory methods

Blood samples were collected before the start of the first hemodialysis session of the week, drawing venous blood into tubes containing trisodium ethylenediamine tetraacetic acid (EDTA) and into plain (dry) tubes. Serum samples were prepared simultaneously and stored until analysis. Complete blood count (CBC), reticulocyte count, and Ret-Hb were measured using the XN-3100™ Automated Hematology Analyzer (Sysmex Corporation, Kobe, Hyogo, Japan). Based on this analyzer's specifications, the reference interval for Ret-Hb was 28-35 pg, and values below 28 pg were considered suggestive of iron deficiency. Biochemical parameters, namely serum ferritin, serum iron, and transferrin, were measured using the Allinity automated analyzer. For defining iron status, we adopted the thresholds recommended by the Kidney Disease: Improving Global Outcomes (KDIGO) guidelines for chronic hemodialysis patients [7]: serum ferritin ≥ 200 ng/mL and transferrin saturation (TSAT) ≥ 20%. The local reference values used by the Biochemistry Laboratory at CHU Mohammed VI were 0.5-1.7 mg/L for serum iron and 0.2-0.4 mg/L for transferrin.

Data analysis

Document preparation (texts, tables, graphs) was done using Microsoft Office Word 2018 (Microsoft Corporation, Redmond, Washington, United States). Data were entered and initially processed using MiniTab software (Minitab, LLC, State College, Pennsylvania, United States). Correlation analyses between variables were performed using linear regression curves and Spearman's correlation coefficient in IBM SPSS Statistics for Windows, Version 26.0 (Released 2019; IBM Corp., Armonk, New York, United States), with statistical significance set at p < 0.05. The diagnostic performance of Ret-Hb for detecting iron deficiency was evaluated using the receiver operating characteristic (ROC) curve method, with the area under the curve (AUC) calculated using MATLAB software (The MathWorks, Inc., Natick, Massachusetts, United States).

Ethical consideration

As per provisions of Article 2 of Moroccan Law 28-13, which governs biomedical research and the protection of participants, the study qualifies for exemption from formal IRB approval as it is an observational study. According to the law, non-interventional or observational research does not require IRB approval unless it involves experimental treatments or interventions.

## Results

Epidemiological data

**Figure 1:**
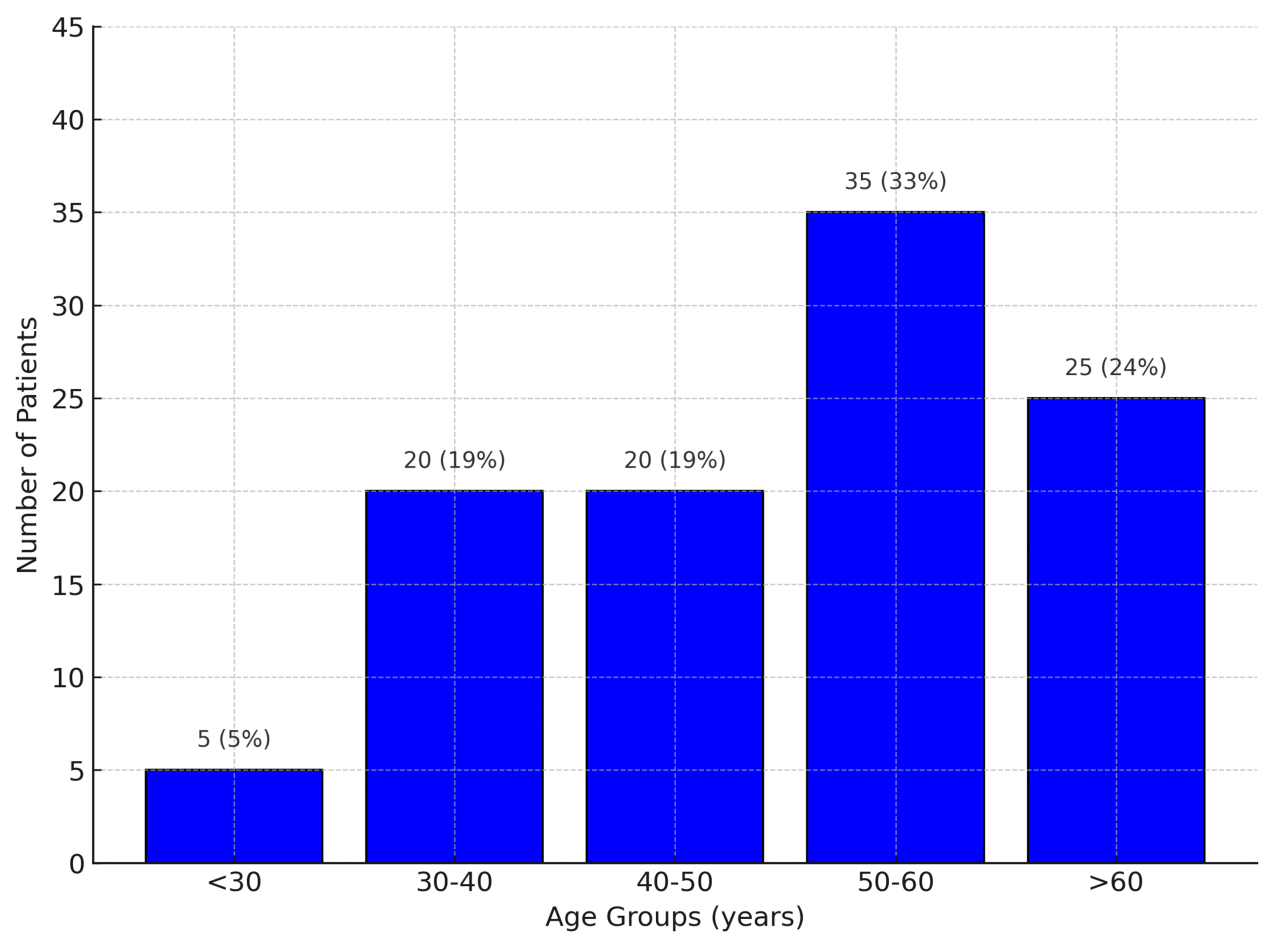
Distribution of patients by age group (N=105)

A total of 105 patients were included in the study. The mean age of the participants was 50.4 ± 16.3 years, ranging from 18 to 80 years. The most represented age group was 50-60 years (Figure 1). There was a female predominance, with 77 female (73.3%) and 28 male (26.7%) participants, corresponding to a male-to-female sex ratio of 0.36.

Hemodialysis characteristics

**Figure 2:**
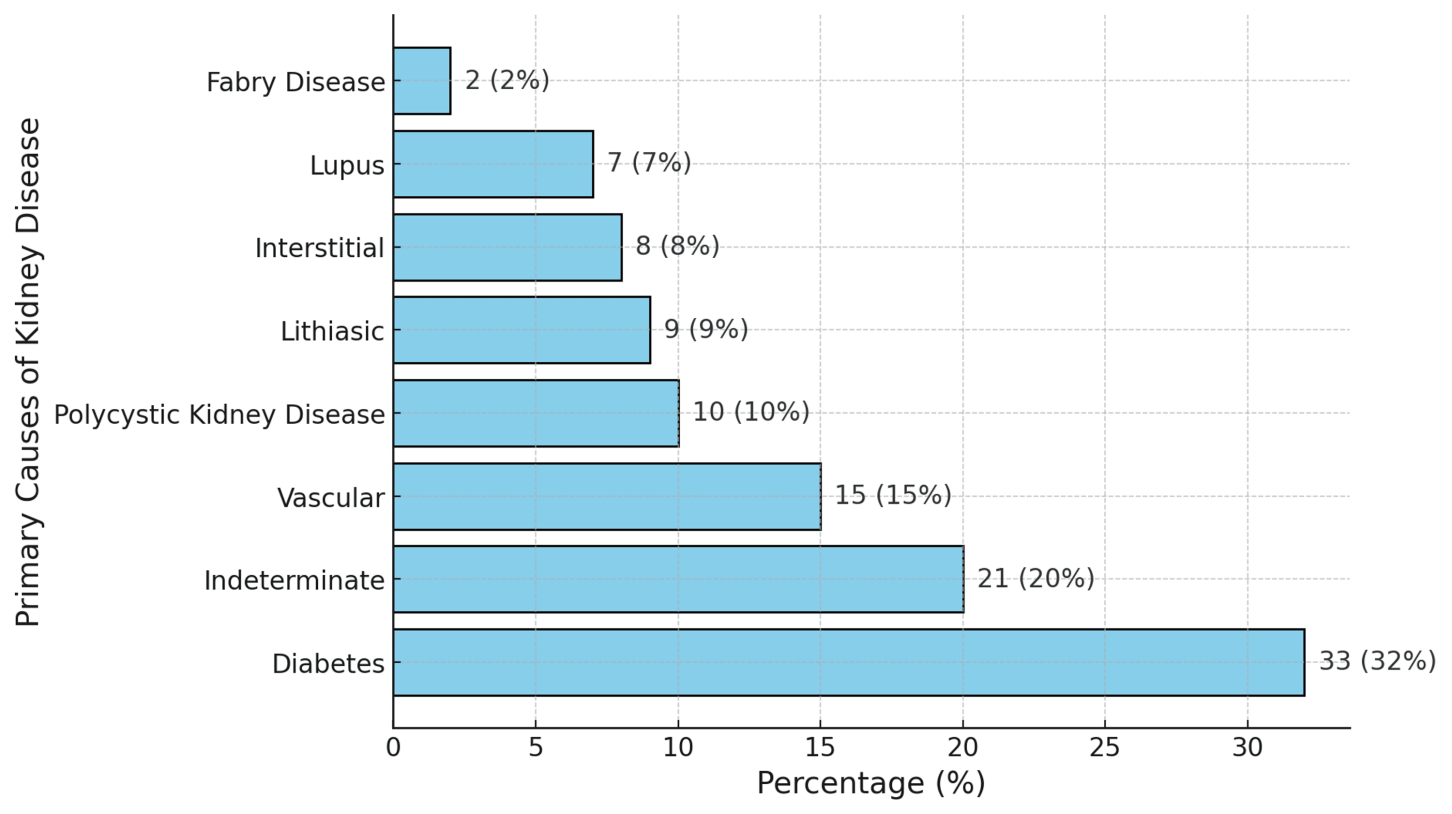
Primary causes of kidney disease (N=105)

The etiology of ESRD was diabetic nephropathy in 32% of cases (Figure 2). The dialysis vintage of the studied patients ranged from three to 302 months, the median was 46 months, with the first quartile Q1 at 24 months and the third quartile Q3 at 109 months (24-109). The prescribed dialysis duration was 12 hours/week for all patients.

Use of iron therapy and EPO

In our study, 52.3% of the studied population was on iron therapy, and 71.4% received EPO.

Biological data

A summary of the biological parameters assessed is provided in Table 1.

**Table 1:** Summary of biological parameters in the study population (N=105) Ret-Hb: reticulocyte hemoglobin content; TSAT: transferrin saturation coefficient

Parameter	Mean ± SD/median (range)	Distribution/Frequency
Hemoglobin (g/dL)	9.8 ± 2.0	Anemia was observed in 87 patients (82.8%)
Type of Anemia (based on CBC)	Not applicable	• Normochromic normocytic: 77 (73.3%) • Hypochromic microcytic: 15 (14.3%) • Macrocytic: 12 (11.4%)
Ret-Hb (pg)	30.0 ± 8.1	• Ret-Hb < 28 pg in 32 patients (30.4%)
Serum Iron (mg/L)	0.52 ± 0.34	• Decreased in 67 patients (63.8%)
Transferrin (g/L)	1.9 ± 0.8	• Normal: 42 (40.0%) • Decreased: 63 (60.0%)
Ferritin (ng/mL)	177 (67.7–513.3)	• <200: 55 (52.3%) • 200–500: 48 (45.7%) • >800: 2 (2.0%)
TSAT (%)	Not applicable	• <20%: 75 (71.4%) • >20%: 30 (28.6%)

Results analysis

ROC Curve

**Figure 3:**
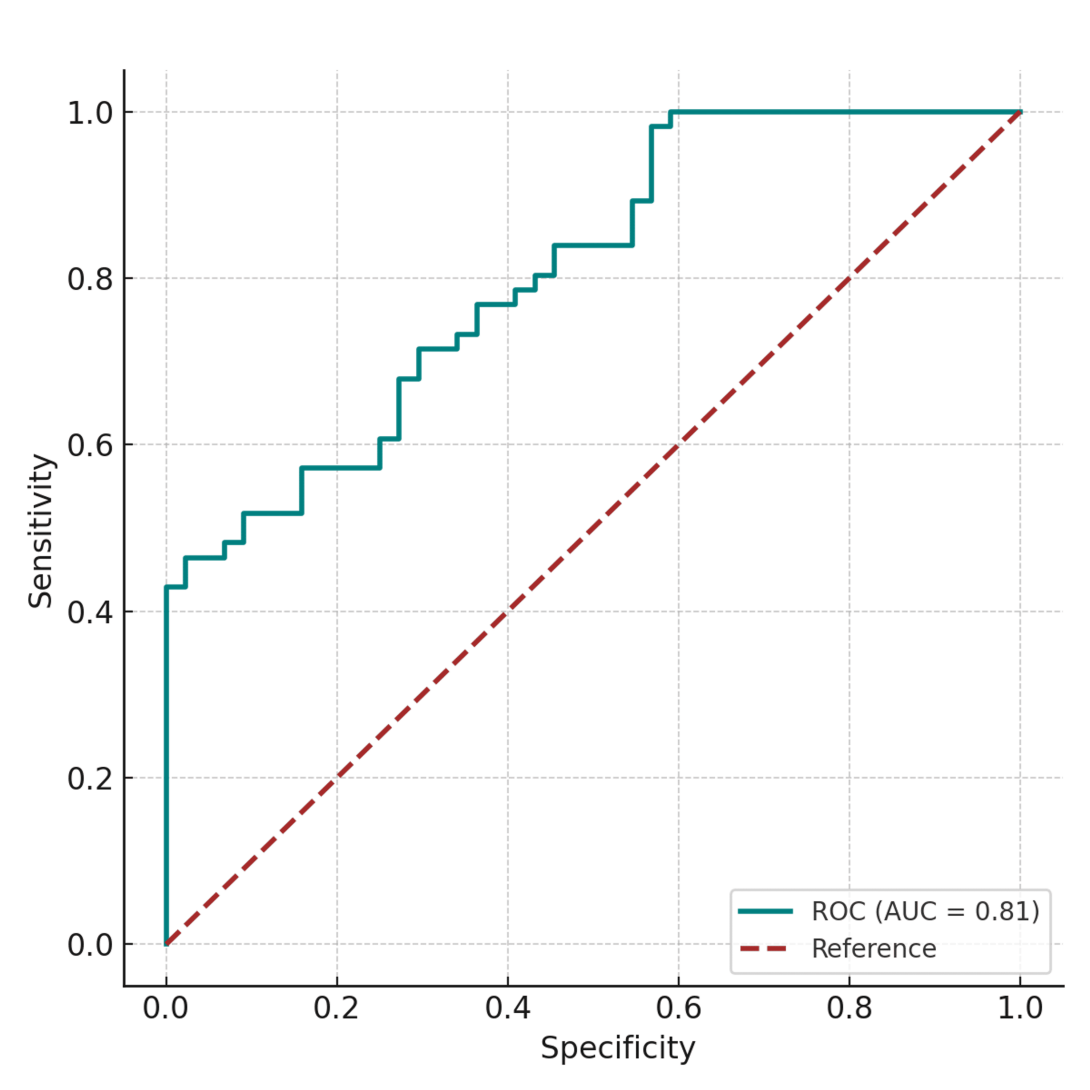
ROC curve for diagnosing iron deficiency using reticulocyte hemoglobin (AUC = 0.81) ROC: receiver operating characteristic; AUC: area under the curve

The ROC curve analysis highlighted the ability of Ret-Hb to diagnose iron deficiency. This was corroborated by a significant area under the curve (AUC) of 0.89, with an optimal threshold set by the Youden index at 25.5 pg (Figure 3). To provide a clearer understanding of the diagnostic performance, the following classifications were made: (i) True Positive (TP): Low Ret-Hb with low transferrin saturation and ferritin levels (correct identification of iron deficiency); (ii) False Positive (FP): Low Ret-Hb with normal transferrin saturation and ferritin levels (incorrect identification of iron deficiency); (iii) True Negative (TN): Normal Ret-Hb with normal transferrin saturation and ferritin levels (correct identification of no iron deficiency); (iv) False Negative (FN): Normal Ret-Hb with low transferrin saturation and ferritin levels (incorrect identification of no iron deficiency). Based on these classifications, Ret-Hb showed a sensitivity of 90% and a specificity of 87.5%.

Correlation between Ret-Hb and Ferritin

**Figure 4:**
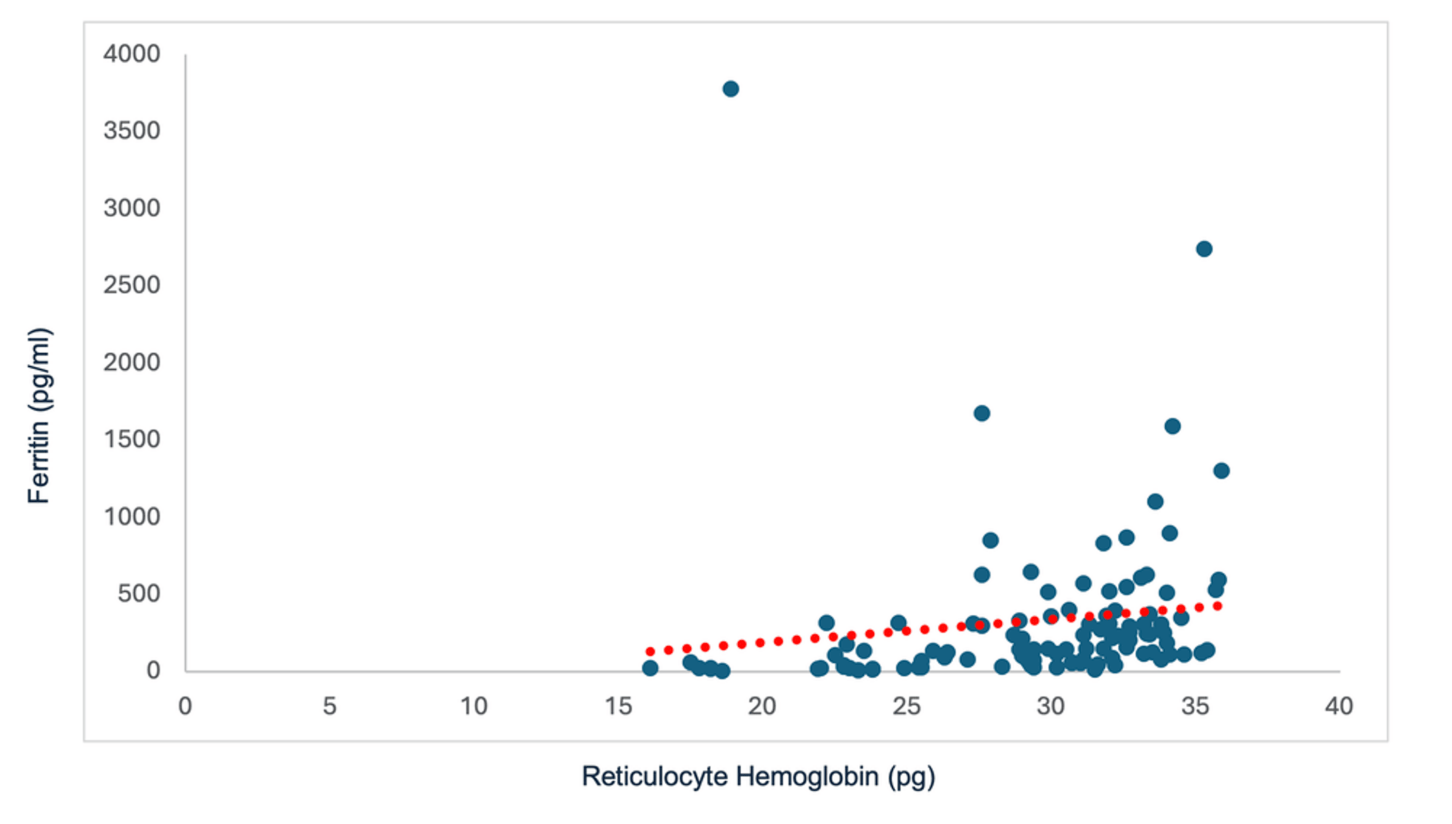
Correlation between Ret-Hb and ferritin Rs = 0.52; p-value = 1.192 x 10^-8^ Ret-Hb: reticulocyte hemoglobin content

The distribution between the two parameters, ferritin and Ret-Hb, showed a moderate positive correlation with a Spearman coefficient of Rs = 0.59 and a p-value = 3.603 x 10^-8^. This correlation was statistically significant (p < 0.0001) and implies that ferritin increased linearly as Ret-Hb increased (Figure 4).

Correlation Between Ret-Hb and Transferrin Saturation Coefficient

**Figure 5:**
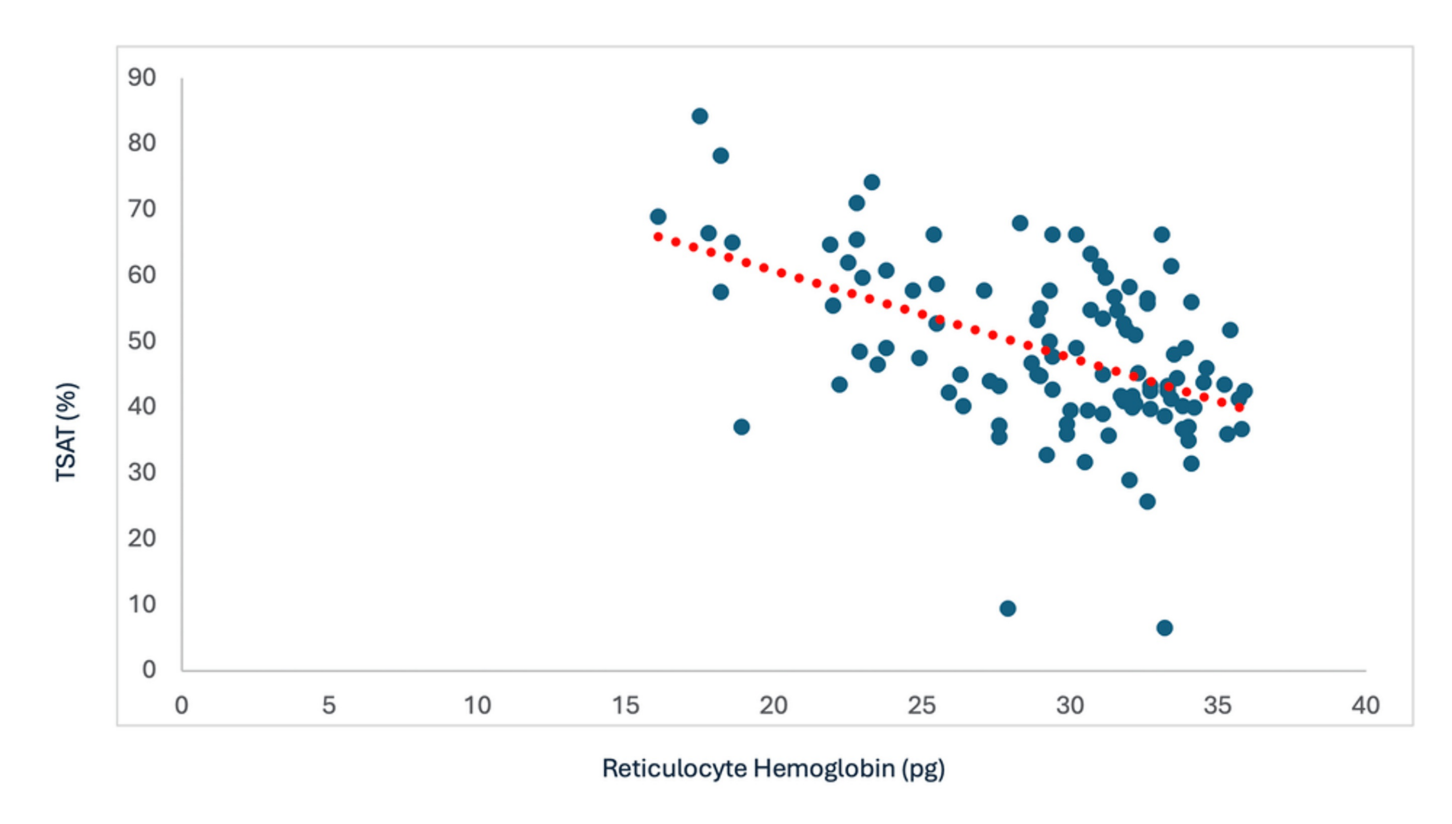
Correlation between Ret-Hb and TSAT Rs = 0.52; p-value = 1.192 x 10^-^^8^ Ret-Hb: reticulocyte hemoglobin content; TSAT: transferrin saturation coefficient

The analysis revealed a moderate positive correlation with a Spearman coefficient of Rs = 0.52 and p-value = 1.192 x 10^-8^; this correlation was therefore statistically significant and implied that as the Ret-Hb level increased, the transferrin saturation coefficient increased linearly (Figure 5).

Analysis of Ret-Hb Level Distribution Based on Iron Therapy and EPO Use

**Figure 6:**
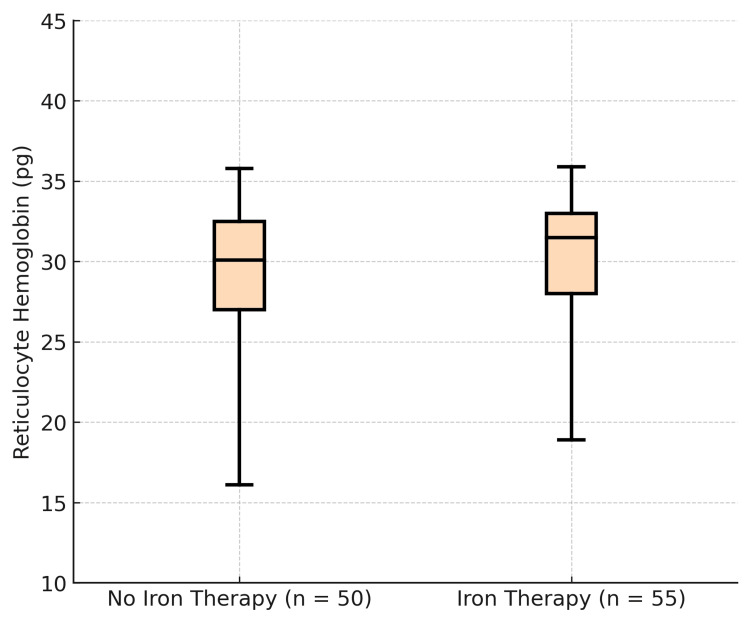
Distribution of Ret-Hb in participants according to iron therapy use (N=105) Ret-Hb: reticulocyte hemoglobin content

The distribution showed Ret-Hb values ranging from 16.1 to 35.8 pg (with a mean of 28.8 pg and a median of 30.1 pg) in patients not receiving iron. For patients who received iron therapy, an increase in Ret-Hb values was recorded, ranging between 18.9 and 35.9 pg (with a mean of 29.7 pg and a median of 31.5 pg) (Figure 6).

**Figure 7:**
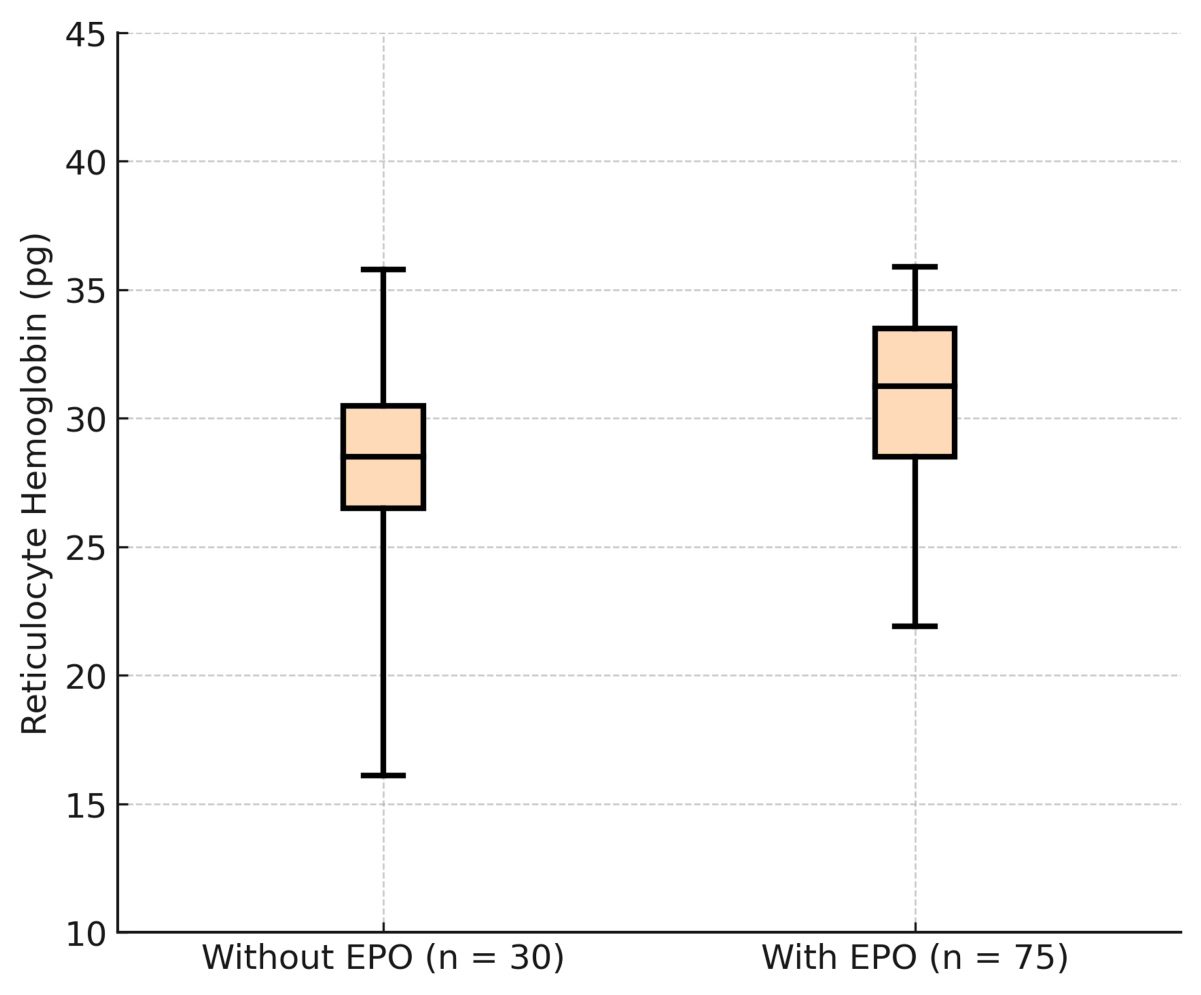
Distribution of Ret-Hb in participants based on EPO therapy (N=105)

Ret-Hb values in patients not on EPO treatment ranged from 16.1 to 35.8 pg, with a mean of 27.92 pg and a median of 28.5 pg. On the other hand, subjects on EPO treatment had Ret-Hb values between 21.9 pg and 35.9 pg, with a mean of 29.85 pg and a median of 31.25 pg (Figure 7).

## Discussion

Anemia constitutes a severe and common complication in CKD, particularly evident in ESRD. It is a public health problem affecting 60-80% of patients with this pathology [4]. It is defined according to the KDIGO recommendation by a hemoglobin concentration below 13 g/dL in men and below 12 g/dL in women [7].

During CKD, iron deficiency and chronic inflammation are two major factors contributing to hyporesponsiveness to erythropoietin treatment [8]. Inflammation, specifically through the increased production of hepcidin, plays a key role in this resistance by mediating iron sequestration. Elevated hepcidin levels inhibit ferroportin, the iron exporter, leading to the internalization and degradation of this protein in macrophages and enterocytes. This process results in the retention of iron within these cells, reducing its availability for erythropoiesis. As a result, despite erythropoietin treatment, iron becomes less available for hemoglobin synthesis, contributing to the observed resistance. In hemodialysis patients, absolute iron deficiency is identified using transferrin saturation levels and serum ferritin measurements [7]. Functional iron deficiency, on the other hand, manifests as sufficient iron stores that are not mobilizable for hemoglobin synthesis. It is therefore difficult to distinguish between inflammatory anemia and mixed anemia (inflammatory and iron-deficient) because, during inflammatory phenomena, standard biological parameters used to assess iron status, such as ferritin or serum iron, can be distorted, making their interpretation delicate [9].

Several parameters have recently become available to assess iron deficiency. The percentage of hypochromic cells (%HYPO) parameter allows detecting a proportion of red blood cells poorly loaded with hemoglobin. Although %HYPO is a direct indicator of iron status, its diagnostic performance can be altered by the lifespan of circulating red blood cells, which can distort the assessment [10]. Another parameter, the concentration of soluble transferrin receptors (sTfR) in circulation, has also been used to diagnose iron deficiency [11]. However, the sTfR level is affected by both iron status and erythropoietic status, meaning that the expression of these receptors increases in states of iron deficiency, while newly formed erythrocytes release many receptors into the circulation [12]. Thus, it would be difficult to distinguish the state of iron deficiency from the expansion of the erythron expressed by newly produced erythrocytes.

Recent advances in automated flow cytometry methods have enabled precise quantitative and qualitative analyses of reticulocytes [13]. Ret-Hb constitutes a sensitive parameter for real-time assessment of iron availability, due to the short lifespan of reticulocytes, which accurately reflects the current conditions of erythropoiesis. The normal concentration of Ret-Hb in adults is 27-35 pg. A Ret-Hb level below 27 pg indicates inefficient erythropoiesis due to iron deficiency, even in the presence of an inflammatory state [5].

Several studies have shown that Ret-Hb remains a stable indicator, little influenced by renal function or hemodialysis sessions. This parameter reflects the efficiency of erythropoiesis in real-time, indicating whether reticulocytes received an adequate amount of iron at the time of their formation. This marker could thus offer an early, precise, and accessible approach for diagnosing iron deficiencies, particularly in cases where classic markers are unreliable. Therefore, based on this new hematological parameter, Ret-Hb, the diagnosis of iron deficiency in cases considered difficult to diagnose using conventional iron indices can be made earlier, with greater precision and low cost, since it is performed on routine hematology analyzers [5].

In this study, we attempted to evaluate the clinical utility of the Ret-Hb parameter as an index of iron status in patients with end-stage renal disease. The mean age of participants was 50.4 ± 16.3 years. In a study conducted in France by Kessler et al., the mean age was 65.6 years [14]. Another study conducted in the United States by Fishbane et al. reported a mean age of 59.6 years [15]. In Cameroon, Kaze et al. observed a mean age of 47.6 years [16]. We noted that in African studies, the mean age is around 50 years, whereas in European and American series, ESRD mainly affects patients in their 6th decade. This may be explained by the age pyramid, with a particularly older population in European and American countries.

Anemia was observed in the majority of participants in the current study, with a percentage of 82.8% and a mean hemoglobin level of 9.8 ± 2.01 g/dl. These results align with the study by Kaze et al. in Cameroon, in which anemia was observed in 79% of the studied population, with a mean hemoglobin of 8.6 ± 1.9 g/dl [16]; and the study by Dalimunthe and Lubis in Indonesia, where anemia was present in 75% of hemodialysis patients with a mean hemoglobin of 8.9 g/dl [17]. The United States Renal Data System (USRDS) 2010 reported that the incidence of anemia in stages 1-4 of CKD is 51.8%, and the mean hemoglobin in ESRD is 9.9 g/dl [18].

The ROC analysis showed diagnostic performance for iron deficiency with a sensitivity approaching 90% and a specificity of 87.5% using a threshold of 25 pg. Consistent with the results of Brugnara and colleagues [13], the current study confirms the diagnostic value of Ret-Hb, with an AUC of 0.913 (P < 0.0001) and a threshold of 27.2 pg, showing a sensitivity of 93.3% and a specificity of 83.2%. In the United Arab Emirates, the study by Sany et al. found good diagnostic performance of Ret-Hb in iron deficiency, with an ROC curve area of 0.887 and a threshold of 27 pg, which observed a sensitivity of 90.4% and a specificity of 80.8% [19]. Another study in Japan by Miwa et al. also observed the performance of Ret-Hb with an AUC of 0.776 and a threshold of 33 pg, showing a sensitivity of 74.3% and a specificity of 64.9% [20].

According to Spearman correlation studies between Ret-Hb and conventional iron parameters, the current study shows a positive linear relationship between Ret-Hb and classic markers of iron status, such as serum ferritin and transferrin saturation coefficient, with notable statistical significance. Our results concur with the study by Dalimunth et al., which observed a moderate positive correlation between Ret-Hb/TSAT and between Ret-Hb/ferritinemia with correlation coefficients of 0.592 (p < 0.0001) and 0.499 (p < 0.0001), respectively [6]. The study by Miwa et al. also found a statistically significant positive correlation between Ret-Hb and TSAT with r = 0.481 and p < 0.01. The correlation coefficient was 0.543 and 0.558 for TSAT < 50% and < 30%, respectively [20]. The analysis revealed a low-magnitude correlation between Ret-Hb and ferritin (r = 0.279 and p < 0.01). The correlation coefficient was 0.309 and 0.355 for serum ferritin < 500 ng/ml and < 100 ng/ml, respectively.

Limitations

This study has limitations. Firstly, its cross-sectional design prevents the establishment of causality or the assessment of Ret-Hb changes over time. Secondly, the generalizability of our study may be limited. By excluding patients with common comorbidities, such as significant inflammation, our study population may not fully represent the clinical complexity typically encountered in hemodialysis patients. This exclusion could potentially restrict the broader applicability of our findings. This limitation related to the patient sample is further compounded by the study being conducted in a single region (Marrakesh). 

## Conclusions

This study highlights the relevance of Ret-Hb compared to other conventional biological markers for detecting and assessing iron deficiency. Ret-Hb shows promise as a reliable and sensitive indicator of iron deficiency in chronic hemodialysis patients. This marker plays a pivotal role in the management of anemia in hemodialysis patients by providing a more accurate evaluation of iron bioavailability in the bone marrow. Moreover, this study proposes a forward-looking approach in the monitoring of anemic hemodialysis patients with low Ret-Hb levels. 

Regular monitoring of Ret-Hb can facilitate the assessment of the patient’s response to iron supplementation, ensuring appropriate adjustments in treatment. The goal of this study is to optimize iron therapy in chronic hemodialysis patients, ensuring that those who would benefit from iron supplementation receive it, while avoiding toxicity from iron overload in those unlikely to benefit. In this context, the integration of Ret-Hb testing into routine clinical practice could significantly enhance personalized care for anemia in hemodialysis patients. Future studies should investigate the long-term clinical benefits of regular Ret-Hb monitoring, its role in predicting anemia progression, and its impact on clinical decision-making. Ultimately, Ret-Hb has the potential to become an essential tool in the precise management of anemia in hemodialysis, reducing the incidence of iron deficiency and improving therapeutic outcomes.
